# The Maternal-to-Zygotic Transition Targets Actin to Promote Robustness during Morphogenesis

**DOI:** 10.1371/journal.pgen.1003901

**Published:** 2013-11-07

**Authors:** Liuliu Zheng, Leonardo A. Sepúlveda, Rhonald C. Lua, Olivier Lichtarge, Ido Golding, Anna Marie Sokac

**Affiliations:** 1 Verna & Marrs McLean Department of Biochemistry and Molecular Biology, Baylor College of Medicine, Houston, Texas, United States of America; 2 Center for Biophysics and Computational Biology, University of Illinois at Urbana-Champaign, Urbana, Illinois, United States of America; 3 Department of Molecular and Human Genetics, Baylor College of Medicine, Houston, Texas, United States of America; 4 Center for Theoretical Biological Physics, Rice University, Houston, Texas, United States of America; 5 Center for the Physics of Living Cells, University of Illinois at Urbana-Champaign, Urbana, Illinois, United States of America; Janelia Farm Research Campus, Howard Hughes Medical Institute, United States of America

## Abstract

Robustness is a property built into biological systems to ensure stereotypical outcomes despite fluctuating inputs from gene dosage, biochemical noise, and the environment. During development, robustness safeguards embryos against structural and functional defects. Yet, our understanding of how robustness is achieved in embryos is limited. While much attention has been paid to the role of gene and signaling networks in promoting robust cell fate determination, little has been done to rigorously assay how mechanical processes like morphogenesis are designed to buffer against variable conditions. Here we show that the cell shape changes that drive morphogenesis can be made robust by mechanisms targeting the actin cytoskeleton. We identified two novel members of the Vinculin/α-Catenin Superfamily that work together to promote robustness during *Drosophila* cellularization, the dramatic tissue-building event that generates the primary epithelium of the embryo. We find that zygotically-expressed Serendipity-α (Sry-α) and maternally-loaded Spitting Image (Spt) share a redundant, actin-regulating activity during cellularization. Spt alone is sufficient for cellularization at an optimal temperature, but both Spt plus Sry-α are required at high temperature and when actin assembly is compromised by genetic perturbation. Our results offer a clear example of how the maternal and zygotic genomes interact to promote the robustness of early developmental events. Specifically, the Spt and Sry-α collaboration is informative when it comes to genes that show both a maternal and zygotic requirement during a given morphogenetic process. For the cellularization of Drosophilids, Sry-α and its expression profile may represent a genetic adaptive trait with the sole purpose of making this extreme event more reliable. Since all morphogenesis depends on cytoskeletal remodeling, both in embryos and adults, we suggest that robustness-promoting mechanisms aimed at actin could be effective at all life stages.

## Author Summary

Every embryo develops under its own unique set of circumstances, with variable inputs coming from mother, father, and the environment. To then ensure a reliable outcome, mechanisms are built into development to buffer against challenges like genetic deficiency, maternal fever, alcohol exposure, etc. This buffering, called *“robustness”*, can be overwhelmed, ending in miscarriage, pre-mature birth, and structural and functional birth defects. Thus, we need to understand how robustness arises in order to define an embryo's susceptibilities to genetic background and environment; and to ultimately promote healthy reproduction. In this work we provide new insight into how morphogenesis, the process of tissue building in embryos, is made more robust. First, we show that early gene expression by the embryo, or zygote, supplements the stockpile of proteins already supplied by the mother to ensure the robustness of early morphogenesis. Specifically, our data suggests that a specific gene, *sry-α*, and its expression by the embryo at the maternal-to-zygotic transition, is a genetic adaptation with the sole function of making the first tissue building event in the fruit fly more robust. In addition, we show that the robustness of this morphogenetic event is promoted by mechanisms regulating the actin cytoskeleton.

## Introduction

Every embryo develops under its own unique set of circumstances and challenges. To then ensure a reliable outcome, mechanisms are built into development to buffer against fluctuations in genetic, biochemical, and environmental inputs [1]. This buffering, called “robustness”, can be overwhelmed, ending in miscarriage, shortened gestation, and structural and functional birth defects [2]. Thus, we need to understand how developmental robustness arises in order to define an embryo's susceptibilities to genetic/epigenetic background and environment; and to ultimately promote healthy reproduction.

Many mechanisms are used to buffer biological systems against fluctuating inputs, including redundant protein function [3], [4], secondary or “shadow” enhancers [5], [6], smart network design [7]–[10], and chaperone activity [3], [11]–[14]. Among developmental systems, a rigorous quantitative understanding of these mechanisms has been largely limited to examples where cell fate decisions are made, and robustness is fostered at the level of gene expression [5], [6], [10], [15] or signaling [9], [16], [17]. For morphogenesis, which translates cell fate decisions into embryonic form, the detailed characterization of specific buffering mechanisms has been slower to come. Morphogenesis requires activities that span nuclei, cytoplasm, and whole tissues, and is driven by cell shape change [18], [19]. So robustness could be promoted at many levels (e.g. gene expression, signaling, membrane dynamics, cytoskeletal remodeling, and cell adhesion). But we do not know enough about the molecular and mechanical underpinnings of morphogenesis to predict where its greatest susceptibilities are, or where buffering mechanisms would be most effective. Specifically, we lack a comprehensive understanding of how cell biological steps convert gene expression into reliable tissue-building events. Actin and microtubules seem like good targets for robustness-promoting mechanisms during morphogenesis because they drive cell-shape change and modulate the mechanics of cells and tissues [20], [21]; however, experimental support for this is lacking.

**Figure 1:**
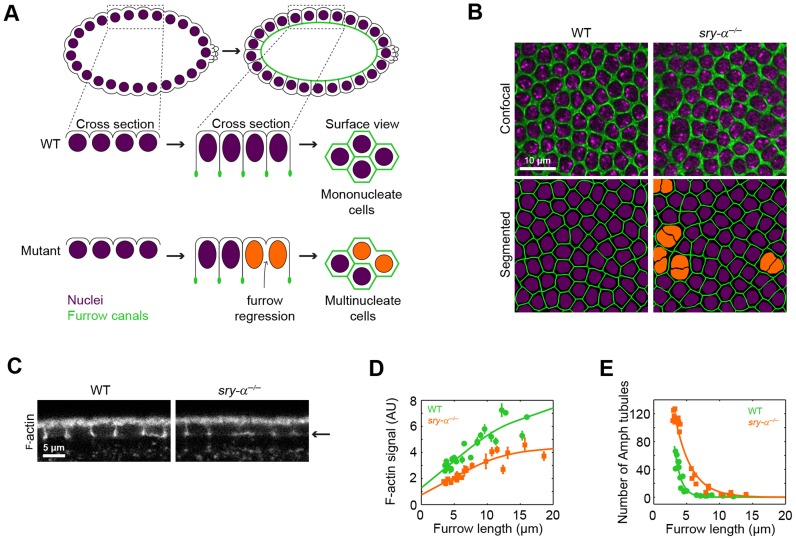
Sry-α regulates cortical F-actin levels. (A) Schematic of cellularization. In wild-type embryos (WT), furrows ingress between nuclei (purple) to form mononucleate cells. In mutants with reduced F-actin, some furrows regress, resulting in multinucleate cells (orange nuclei). (B) Surface views of furrow canals stained for Myosin-2 in embryos of indicated genotypes. Multinucleate cells highlighted (orange nuclei) in corresponding segmented images. (C) Cross sections show F-actin levels, as detected by phalloidin staining. Arrow indicates furrow canal position. (D) Quantification of F-actin levels in furrow canals. Each point represents one embryo with ≥75 furrow canals measured (mean ± s.d.). (E) Quantification of Amphiphysin (Amph) tubules. Each point represents one embryo with ≥100 furrows analyzed.

In order to identify the mechanisms that promote robustness, the outcomes of the process in question must be quantifiable [1]. For example, we know of robustness-promoting mechanisms for cell fate decisions in development because the outcomes are binary, and typically happen along well-separated spatial dimensions so that fidelity can be readily tracked and quantified over a range of perturbations [5], [6], [9], [10], [15]–[17]. For morphogenesis, which is spatially complex, challenging to image, and has long been scored by qualitative rather than quantitative methods, fidelity is not easily measured. Consequently, the number of well-tested examples that show how robust morphogenesis is achieved remains low, in the context of both individual cell shape change and whole tissue remodeling [22], [23]. To address this gap in our knowledge, we are using the first tissue-building event in the fly embryo, cellularization, as a simple, quantifiable model to study robustness. Fly embryos first develop as a syncytium, passing through 13 mitoses with no intervening cytokinesis. Then, during cell cycle 14 the embryo undergoes cellularization, during which ∼6000 cortically-anchored nuclei are simultaneously packaged into a sheet of cells that will be the primary epithelium (Figure 1A) [24]. Cellularization takes ∼60 minutes and plasma membrane furrows ingress 35 µm, cutting straight between adjacent nuclei to form mononucleate cells. This simple architecture allows unambiguous quantification of the fidelity of cellularization, where furrow failures or regressions show up as multinucleate cells, and hundreds to thousands of packaging events can be assayed per embryo to generate a ratio of mononucleate cells-to-nuclei (ratio = 1 in wild-type embryos; Figure 1A).

Fly embryos cellularize just after the maternal-to-zygotic transition (MZT) [24], when transcription from the zygotic genome starts to maximally impact the developmental program [25]. Thus, the few zygotic genes that are required for cellularization have long been thought of as “switches” to control this morphogenetic event [26], [27]. However, we now report a new role for one of these long-supposed switches, Sry-α. We identify Sry-α as a zygotic gene product that is expressed at the MZT, not to control cellularization, but rather to make it robust in the face of both environmental and genetic perturbations. We find that Sry-α acts together with its maternally provided paralog Spt to reinforce the actin cytoskeleton and so promote robust cellularization. Our data provides a clear example of how zygotic contributions, made at the MZT, not only instruct development, but also supplement the maternal machinery to ensure the fidelity of specific morphogenetic events. What's more, our data suggests that this robustness is fostered via regulation of the actin cytoskeleton.

## Results

### Sry-α regulates F-actin levels during cellularization

At the start of cellularization, actin filaments (F-actin) accumulate at incipient furrow tips, which in surface views form furrow “canals” around the nuclei (Figure 1A) [24]. Furrow canals are then maintained throughout cellularization, and are required for stable furrow ingression (Figure 1A) [28]–[30]. We previously showed that mutations or drug treatments that reduce F-actin levels in all furrow canals, precipitate the regression of a fraction of furrows (Figure 1A) [29], [30], consistent with multinucleation phenotypes reported for actin regulators like Rho1 GTPase, RhoGEF2, and the Formin, Diaphanous [28], [31], [32]. In an effort to identify other actin regulators that are required for the fidelity of cellularization, we examined a poorly characterized mutant, *serendipity-α* (*sry-α*), that similarly displays a multinucleation phenotype (Figure 1B) [26], [27]. The *sry-α* gene was previously mapped, and is expressed at the MZT just prior to cellularization [27]. Consequently, *sry-α* has long been thought of as a developmental cue that provides some new activity to trigger cellularization [26], [27]. We found that all furrow canals in *sry-α* null mutants (*sry-α^−/−^*) have significantly reduced levels of F-actin compared to wild-type, throughout cellularization (Figure 1C, 1D). In addition, incipient furrow canals in *sry-α^−/−^* mutants display an increased number of Amphiphysin tubules (Figure 1E), which indicates promiscuous endocytosis upon F-actin reduction [29], [33]. These results show that Sry-α regulates F-actin levels in furrow canals during cellularization.

### Sry-α and Spt represent a novel clade of the Vinculin/α-Catenin Superfamily

**Figure 2:**
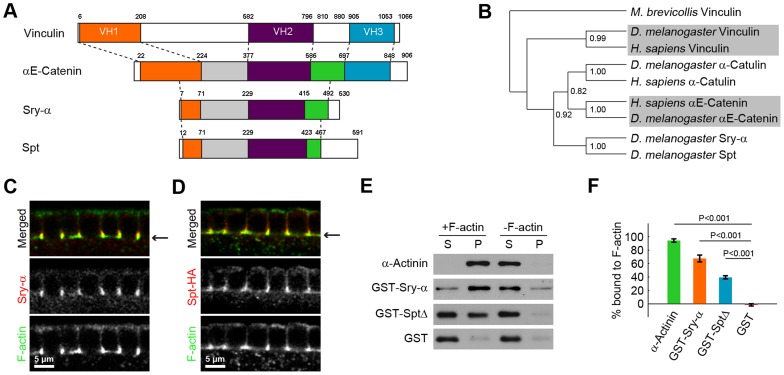
Sry-α and Spt are paralogs with redundant F-actin binding activity. (A) Domain structure of Vinculin/α-Catenin Superfamily members. Vinculin Homology domains 1, 2, and 3 (VH1, VH2, VH3) are orange, purple, and blue, respectively. The green and gray domains indicate sequences shared between α-Catenin, Sry-α, and Spt. White areas indicate unique sequences. Dashed lines are guides to show how the different proteins align with each other. (B) Cladogram of Vinculin/α-Catenin Superfamily. Bootstrap statistics at branch points (1000 iterations). Outgroup is *M. brevicollis* Vinculin. Shading highlights division of the clades. (C, D) Cross sections show (C) Sry-α and (D) Spt-HA co-localization with F-actin in furrow canals. Arrows indicate furrow canal position. (E) Western blots for an F-actin co-sedimentation assay show Sry-α full-length protein (GST-Sry-α) and Spt truncate (GST-SptΔ; amino acids 250–461) pellet with F-actin. α-Actinin and GST are positive and negative controls, respectively. S, supernatant; P, pellet. (F) Quantification of percent proteins bound to F-actin from three independent co-sedimentation assays (mean ± s.e.m.). Student's t-test was performed to calculate P values as shown.

Based on remote homology searches, including PHYRE [34] and I-TASSER [35], we found that Sry-α is a novel member of the Vinculin/α-Catenin Superfamily (Figure 2A, 2B) [36]. Our analysis also identified a Sry-α paralog in the *D. melanogaster* genome that we called Spitting Image (Spt, CG8247). Sry-α and Spt align with the middle sequences of Vinculin and α-Catenin, including the Vinculin-Homology 2 domain (VH2; Figure 2A, Figure S1) [37], [38]. Like α-Catulin, Sry-α and Spt represent a distinct clade of the Vinculin/α-Catenin Superfamily (Figure 2B, Figure S2) [39], [40]. Based on a “roll-call” analysis of orthologs in organisms with fully sequenced genomes, *sry-α* and *spt* co-exist in all Drosophilids, while *spt* alone is present in other insects (see Table S1). In higher metazoans, PHYRE analysis also identified other uncharacterized proteins, like Sry-α and Spt, which share remote homology with the middle sequences of Vinculin and α-Catenin (Figure S1, S2). Members of the Vinculin/α-Catenin Superfamily peripherally associate with the plasma membrane and interact with the actin cytoskeleton [36]. Thus, this evolutionary relationship is functionally consistent with a role for Sry-α and Spt at the actin cortex during cellularization.

### Paralogs Sry-α and Spt have redundant actin-regulating activity

To examine the relationship between Sry-α and Spt, we asked if these paralogs have unique or overlapping functions. Both Sry-α and HA-tagged Spt localize to F-actin rich furrow canals in cellularizing embryos (Figure 2C, 2D). In addition, we used an F-actin co-sedimentation assay to show that both Sry-α and Spt bind F-actin directly. Recombinant Sry-α and Spt proteins were purified from insect cells and mixed with F-actin. Upon centrifugation, F-actin and its interacting proteins pellet (α-Actinin; Figure 2E, 2F), while unbound proteins remain in the supernatant (GST; Figure 2E, 2F). For both Sry-α and Spt, we detected a significant fraction of F-actin bound protein (Figure 2E, 2F). Thus, the co-localization of Sry-α and Spt, as well as their biochemical activity, suggest that they could act redundantly during cellularization.

**Figure 3:**
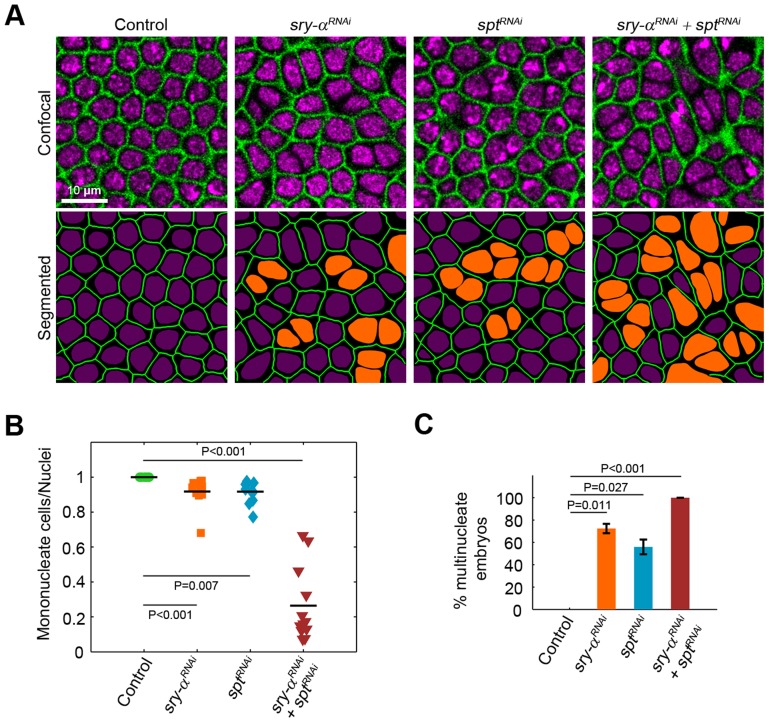
*sry-α* and *spt* induce the same loss of function phenotype. (A) Surface views of furrows and nuclei, detected in living embryos (membrane Gap43-mCherry, green; nuclear Histone-GFP, purple), following the indicated *sry-α^RNAi^* or *spt^RNAi^* treatment. Multinucleate cells highlighted (orange nuclei) in corresponding segmented images. (B) Quantification of multinucleation phenotypes. Each point represents one embryo with ≥150 nuclei analyzed (n = 12 embryos per condition). (C) Frequency of multinucleation for embryos following the indicated RNAi treatment (n≥6,000 nuclei from 30 embryos per treatment; mean ± s.e.m.). Student's t-test was performed to calculate P values as shown in (B, C).

**Figure 4:**
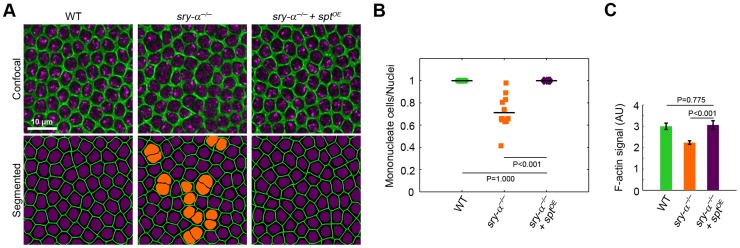
Sry-α and Spt share redundant function during cellularization. (A) Surface views of furrow canals stained for Myosin-2 in embryos of indicated genotypes. Multinucleate cells highlighted (orange nuclei) in corresponding segmented images. (B) Quantification of multinucleation phenotypes. Each point represents one embryo with ≥150 nuclei analyzed (n = 12 embryos per condition). (C) Quantification of F-actin in furrow canals of furrows of length 3–5.5 µm (n = 9 embryos per genotype, with 15 furrows analyzed per embryo; mean ± s.e.m.). Student's t-test was performed to calculate P values as shown in (B, C).

To look for overlapping *in vivo* functions, we first used RNAi [41] and found that *spt* knockdown (*spt^RNAi^*) causes multinucleation during cellularization, which is qualitatively indistinguishable from either the *sry-α^−/−^* genetic mutant or *sry-α* RNAi (*sry-α^RNAi^*; Figure 3, Figure S3). Additionally, embryos with double *spt^RNAi^* plus *sry-α^RNAi^* knockdown display a strongly enhanced multinucleation phenotype (Figure 3B, 3C). Together, the localization, biochemistry, and RNAi phenotypes suggest that Sry-α and Spt share redundant functions during cellularization. To then confirm this, we tested whether Spt overexpression (*spt^OE^*) can rescue *sry-α^−/−^* multinucleation. We used Sry-α immunostaining to genotype embryos (Figure S4A), and found that 100% of *sry-α^−/−^* mutants are rescued by *spt^OE^* (Figure 4A, 4B, Figure S4). Rescue of *sry-α^−/−^* multinucleation by *spt^OE^* is equivalent to that accomplished with a genomic construct encoding *sry-α* itself (Figure S4B). We also confirmed that *spt^OE^* restores F-actin to wild-type levels in *sry-α^−/−^* mutants (Figure 4C). Thus, Sry-α and Spt share an overlapping actin regulating function during cellularization.

### Spt is maternally provided while Sry-α is expressed zygotically

**Figure 5:**
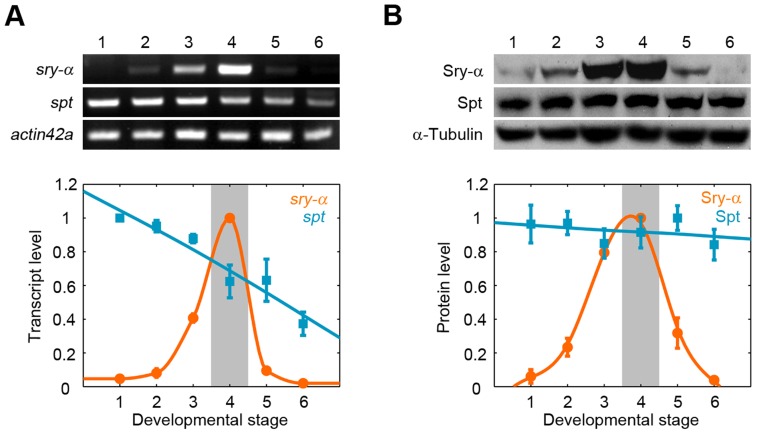
Spt is maternally provided, while Sry-α is zygotically expressed in a pulse for cellularization. (A) RT-PCR and (B) Western blots show Sry-α and Spt expression profiles. Developmental stages are (1) mitotic cycles 1–9; (2) mitotic cycles 10–11; (3) mitotic cycles 11–13; (4) cellularization (shaded); (5) ventral furrow formation; (6) segment formation. Profiles were obtained from three independent RT-PCR or Western Blotting experiments (mean ± s.e.m.).

These results challenge a long-standing idea that *sry-α* is zygotically expressed at the MZT to supply some new activity that instructs cellularization to proceed [26], [27]. In fact, the Sry-α activity may already be available via Spt. We compared the developmental expression profiles of endogenous Sry-α and Spt. As previously shown, *sry-α* transcript and protein are expressed at the MZT, in a pulse that just coincides with cellularization (Figure 5A, 5B) [27]. Conversely, *spt* transcript and protein are provided maternally, and Spt levels persist throughout and far beyond cellularization (Figure 5A, 5B, Figure S5). That is, a pulse of zygotically expressed Sry-α adds to a pool of maternally provided Spt during cellularization. (i.e. Sry-α expression only boosts an already existing Spt activity in the embryo.) Given that Spt can completely replace Sry-α during cellularization (Figure 4, Figure S4), Sry-α does not supply some unique activity that triggers the process. Instead, the expression profiles suggest that the level of Spt plus Sry-α is somehow critical for the successful progression of cellularization.

### Spt plus Sry-α work together to promote robust cellularization

**Figure 6:**
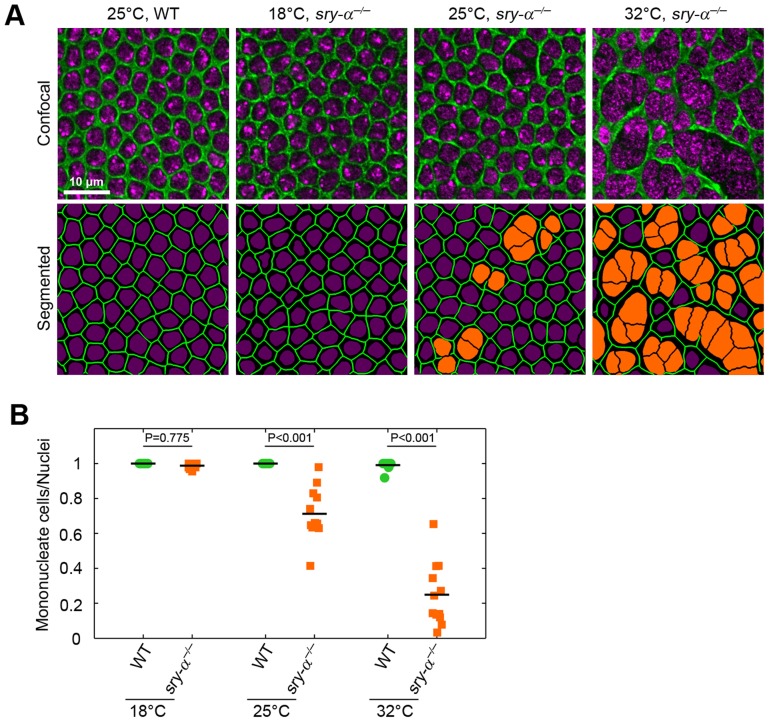
Sry-α is not required for cellularization at an optimal temperature of 18°C, but is required as conditions approach an extreme of 32°C. (A) Surface views of furrow canals stained for Myosin-2 in embryos of indicated genotypes. Multinucleate cells highlighted (orange nuclei) in corresponding segmented images. (B) Quantification of multinucleation phenotype for indicated genotype and temperature. Each point represents one embryo with ≥150 nuclei analyzed (n = 12 embryos per condition). Two-way ANOVA analysis was performed to calculate P values as shown.

It was previously shown that the co-expression of paralogs in worms and yeast promotes biological robustness [3], [4]. Presumably, these “gene duplicates” provide overlapping functions, and so replace or supplement each other in the face of internal and external perturbations [3], [4]. Hence, we hypothesized that there is a threshold level of Sry-α plus Spt activity that is required for cellularization to proceed with high fidelity. This robustness hypothesis predicts that at an optimal condition, Sry-α function is dispensable and Spt alone can support successful cellularization, whereas both are needed at sub-optimal conditions. To test this, we assayed for multinucleation phenotypes in *sry-α^−/−^* null mutants at an optimal temperature. We chose 18°C, the lowest temperature at which *D. melanogaster* thrives. We reasoned that the lower temperature would reduce the demand on the actin cytoskeleton, because F-actin is more stable at lower temperature [42]. As predicted, we found that multinucleation is suppressed in *sry-α^−/−^* mutants that are reared at 18°C (Figure 6A, 6B). At this temperature, the ratio of mononucleate cells to nuclei in *sry-α^−/−^* mutants is not significantly different than wild-type (Figure 6B). Nor did we detect changes in the dimensions of the cells that formed for *sry-α^−/−^* mutants at 18°C (data not shown). Thus, Spt activity is sufficient for cellularization to proceed at an optimal condition. However, multinucleation in *sry-α^−/−^* mutants was increasingly severe at higher temperatures (25–32°C; Figure 6A, 6B), showing that Spt is not enough to ensure reliable cellularization when conditions deviate from the optimal.

**Figure 7:**
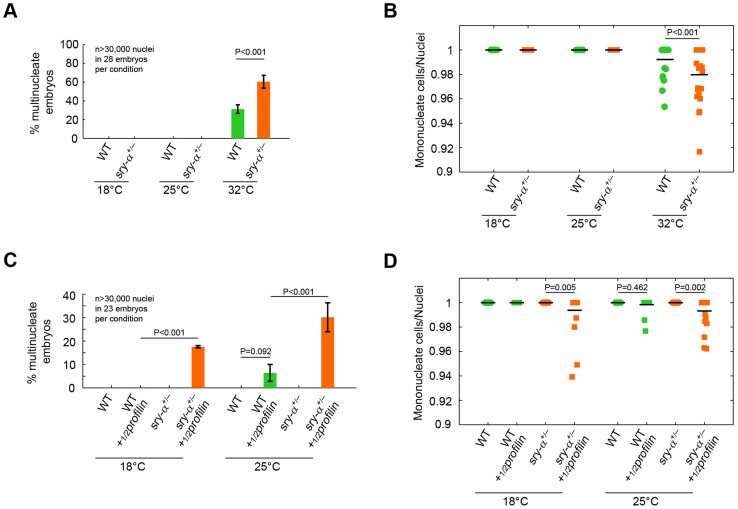
Two copies of *sry-α* are required for reliable cellularization at the extreme temperature of 32°C or when the embryo is challenged by a genetic perturbation. (A, C) Frequency of multinucleation for heterozygous embryos reared (A) at 32°C, or (C) with a reduced dose of maternal Profilin (*½profilin*; mean ± s.e.m.). (B, D) Quantification of multinucleation phenotype for heterozygous embryos reared (B) at 32°C, or (D) with a reduced dose of maternal Profilin (*½profilin*). Each point represents one embryo with ≥150 nuclei analyzed (n = 23 embryos per condition). Two-way ANOVA analysis was performed to calculate P values as shown in (A–D).

A second prediction of the robustness hypothesis is that reducing *sry-α* dosage will make cellularization more likely to fail when the embryo is challenged by perturbations [3], [4]. To test this, we reduced the Sry-α level using genetic heterozygosity, and looked for multinucleation at an extreme temperature of 32°C. This temperature marks an upper limit at which *D. melanogaster* embryos can survive, but developmental events are measurably impaired [5], [6]. We found that the occurrence of multinucleation at 32°C is significantly increased for *sry-α* heterozygotes (*sry-α^+/−^*; Figure 7A, 7B). Genotypes were confirmed by RNA FISH (Figure S6) [43]. So while Spt alone is sufficient for cellularization at an optimal temperature, Spt plus two copies of the *sry-α* gene are required when environmental conditions are extreme. This suggests that the expression of Sry-α serves as a robustness-promoting mechanism for cellularization.

A hallmark of robustness-promoting mechanisms is that they respond equally well to different kinds of perturbations (e.g. genetic, biochemical, or environmental) [44]. For example, shadow enhancers for the fly genes *snail* and *shavenbaby* promote robust gene expression at high temperature, and when mutations reduce input from their respective activation pathways [5], [6]. In addition, a systems-level analysis in yeast showed that genes that promote mutational robustness also promote high fitness in a wide range of stressful environments [44]. Thus, we also tested whether the Sry-α level ensures the fidelity of cellularization upon genetic perturbation. We checked for multinucleation in *sry-α^+/−^* heterozygotes that carry only a half maternal dose of *profilin* (*½profilin*). Profilin is an actin accessory protein that promotes actin polymerization [45]. Strikingly, we saw multinucleation in *sry-α^+/−^* heterozygotes in the *½profilin* background even at 18°C (Figure 7C, 7D). We conclude that the fidelity of cellularization, in the face of both environmental and genetic perturbations, critically depends on the level of Sry-α.

## Discussion

Our data support a model wherein maternal Spt plus zygotic Sry-α work together to promote the robustness of cellularization. Our findings refute the idea that there is a clear passing off of developmental control from the maternal to the zygotic machinery at the MZT [25]–[27], [46]. For example, the maternal genome was previously thought to provide the basic cellular machinery for cellularization, while Sry-α and other zygotic players provided the instructions [26], [27]. More recently zygotic gene products have been shown to actively degrade maternal mRNAs, arguing that there may be a clean break from maternal to zygotic control [47]–[49]. In both cases, the zygotic contribution is largely viewed as being instructive. But our data speaks to a more collaborative interaction between the maternal and zygotic genomes: We show that cellularization can proceed with no input from zygotic Sry-α. Instead of controlling cellularization, we find that Sry-α actually adds to the activity of maternal Spt to make this morphogenetic event more reliable in the face of environmental and genetic perturbation. Sry-α is not taking over for the maternal product because it is only expressed in a pulse during the demanding event of cellularization, while maternal Spt persists far beyond cellularization. So, the relationship of Spt and Sry-α illustrates with exceptional clarity that the maternal and zygotic genomes also work together, with redundant activities, to make development robust. Certainly, this collaboration is likely to be broadly conserved (e.g. maternal plus zygotic contributions of Rac proteins in *C. elegans* and Cadherins in *Xenopus* support the progression of specific morphogenetic events [50], [51]).

Since maternal RNAs and proteins are loaded into oocytes and eggs long before they act in development (up to months) [25], their levels may not be very reliable [46]. Overlapping activities encoded by the zygotic genome could then buffer this variability and ensure successful progression through early embryogenesis. In our case, this relationship was revealed by the distinct expression profiles of paralogs provided from the maternal and zygotic genomes. But the same end is likely accomplished by expressing a single gene both maternally and zygotically. In fact, there is the recurrent observation that some proteins are expressed zygotically, while a significant maternal pool still persists (e.g. *Drosophila* β-Catenin) [46], [52]. In *D. melanogaster*, roughly 5% of the genome displays this expression profile, with a maternal contribution supplemented by zygotic expression at the MZT [47].

But why split the contribution between the maternal and zygotic genomes? For cellularization, why is the same level of robustness not achieved by simply expressing more maternal Spt? We can envision at least two possibilities: either Spt and Sry-α have some distinct functions, perhaps at different developmental stages; and/or high levels of Spt/Sry-α protein are harmful.

Sry-α and its expression at the MZT may be an adaptive genetic trait with its function, perhaps its sole function, serving to buffer cellularization against external and internal perturbations. According to our roll-call analysis, both *spt* and *sry-α* are present in the genomes of all Drosophilids, while other insects only encode *spt* (see Table S1). Some aspect of development specific to *Drosophila* may then stabilize the strategy of maternal Spt plus zygotic Sry-α. For example, cellularization in *Drosophila* builds tall columnar cells around thousands of nuclei, and so may be more demanding than cellularization in other insects where shorter cuboidal cells form around fewer nuclei [53]–[55]. Alternatively, Drosophilids share a fast rate of embryogenesis in comparison to many other insects [53], [54], [56], which could make their cellularization more difficult. Thus, the pulse of zygotic Sry-α may be advantageous for meeting the challenge of a very extreme cellularization event in *Drosophila*.

Our data suggests that zygotic Sry-α adds to the activity of maternal Spt to regulate actin, and so ensure the fidelity of cellularization. A significant future challenge will be understanding what specific actin-based activities promote robust morphogenesis. For example, F-actin is a critical determinant of tissue mechanical properties during development because it assembles with Myosin-2 and other crosslinkers, within single cells, to form a cortical network that (1) governs the rigidity and shape of the whole embryo [57]–[59]; and (2) generates the forces for and resistance to the cell shape changes that drive morphogenesis [60]–[64]. Thus, actin could promote robustness by buffering the mechanical properties of cells and tissues. In fact, Spt and Sry-α bind F-actin directly. Also, Spt and Sry-α are related to the F-actin crosslinker Vinculin, and they contain the conserved M-domain at the VH2, which in Vinculin dimerizes to support F-actin crosslinking [38]. So Spt and Sry-α could promote robust cellularization by crosslinking F-actin and modulating tissue mechanics. Alternatively, F-actin also controls the membrane remodeling that accompanies cell shape change and morphogenesis. Specific to *Drosophila* cellularization, F-actin antagonizes endocytosis to favor plasma membrane growth and furrow ingression [29], [33]. Consequently, F-actin could also promote robustness by controlling the membrane dynamics of morphogenesis. Since all morphogenesis depends on actin remodeling, both in embryos [65] and adults [66], we believe that robustness-promoting mechanisms that target actin are likely to be ubiquitous.

## Materials and Methods

### Fly stocks and genetics

The *sry-α^−/−^* and *sry-α^+/−^* embryos were collected from *Df(3R)X3F/TM3*, *Sb* [26] crossed to OreR wild-type flies. The *nulloX* embryos were collected from *C(1)DX*, *ywf* [29], [30]. For *sry-α^−/−^* plus *spt^OE^*, first *matαtub-GAL4* (II) was crossed with *UASp-Spt-HA* (III) (this paper) to make stock *matαtub-Gal4; UASp-Spt-HA*. Second, these flies were crossed with *Df(3R)X3F/TM3*, *Sb*, and finally embryos were collected from *matαtub-GAL4/+; UASp-Spt-HA/Df(3R)X3F* mothers crossed with their sibling *matαtub-GAL4/+; UASp-Spt-HA/Df(3R)X3F* fathers. For *sry-α^−/−^* plus *sry-α^rescue^*, *Df(3R)X3F/TM3*, *Sb* was crossed to *sry-α* genomic rescue stock (II) (gift of E. Wieschaus). For *½profilin*, *chic^221^ cn^1^/CyO; ry^506^* (Bloomington Stock Center #107932) was crossed with *Df(3R)X3F/TM3*, *Sb*; and embryos were collected from *chic^221^ cn^1^/+; Df(3R)X3F/ry^506^* mothers crossed with their sibling fathers *chic^221^ cn^1^/+; Df(3R)X3F/ry^506^*. For RT-PCR and Western Blotting, OreR was used. For RNAi imaging, embryos were injected from stock *Gap43-mCherry/CyO*; *Histone-GFP/TM3*, *Sb* (parental stocks gifts of A. Martin and E. Wieschaus).

### Production of Spt transgene and antibody

For *UASp-Spt-HA* flies, the coding sequence of *D. melanogaster spt* (CG8247) was fused with a C-terminal hemagglutinin (HA) tag, and cloned into pP(UASP) vector. Transgenesis and mapping followed standard methods (BestGene, Inc.). For anti-Spt antibody, the coding sequence of *spt* fused with a C-terminal histidine tag was cloned into pET vector, and recombinant protein was purified from *E. coli*. Antibodies were produced in guinea pigs according to standard methods (Panigen, Inc.). According to the publicly available data on FlyBase (flybase.org), *spt* is not expressed in adult heads. Thus, adult heads served as the negative control for antibody characterization (Figure S5).

### F-actin co-sedimentation assay

Recombinant proteins GST-Sry-α (full length) and GST-SptΔ (amino acids 250–461) were produced in Sf9 cells (Baylor College of Medicine Baculovirus Core/Proteomics Shared Resource). The Spt truncate was used because full-length protein is insoluble in both bacterial and insect expression systems. Proteins were purified with Glutathione Sepharose (GE Healthcare) and dialyzed in F-buffer (20 mM Tris-HCl pH 7.5, 2 mM DTT, 2.5 mM MgCl_2_, 75 mM KCl and 10 mM NaCl). α-Actinin (Cytoskeleton, Inc.) was used as the positive control, and GST for the negative control was purified from Sf9 cells.

Rabbit muscle monomeric actin (G-actin) was extracted from Rabbit Muscle Acetone powder (Pel-Freez Biologicals). 20 mM G-actin was maintained in G-buffer (2 mM Tris-HCl pH 8.0, 0.2 mM ATP, 0.5 mM DTT, and 0.2 mM CaCl_2_) until polymerization in F-buffer by adding buffer and salt and incubating at room temperature for 1 hour. For the co-sedimentation assay, purified proteins were pre-cleared by centrifugation at 900,000 rpm for 30 minutes at 4°C to remove any precipitate. Pre-cleared proteins (∼1 µg) were incubated with F-actin for 30 min on ice and then centrifuged at 900,000 rpm for 30 min at 4°C. Supernatant was removed, and replaced by an equal volume of 3X sample buffer to resuspend pellets. Equal volumes of supernatants and pellets were separated on 5–10% SDS-PAGE gels and transferred to nitrocellulose. GST-Sry-α, GST-SptΔ, and GST were detected by 1∶10,000 mouse anti-GST (ab19256, Abcam). α-Actinin was detected by 1∶1000 anti-α-Actinin (A7811, Sigma-Aldrich). The goat secondary antibodies were HRP conjugates used at 1∶10,000 (Jackson ImmunoResearch).

### Embryo collection and temperature incubations

Embryo collection cups were set up on apple juice plates according to published protocols [29], [30], [41]. For specific temperature incubations, collection cups were housed in air incubators of 18°C, 25°C, or 32°C within a range of ±1°C for 5, 4, or 3 hours, respectively. Plates were harvested and embryos fixed immediately.

### Embryo fixation, staining, and FISH

For immunofluorescence, embryos were fixed in boiling salt buffer; or 4% paraformaldehyde, 0.1 M phosphate buffer (pH 7.4): heptane (1∶1) [29]. Antibody concentrations and fixation methods are listed in Table S2. Due to Myosin-2 antibody incompatibility with FISH, Septin (Peanut) was used as the furrow canal marker for experiments where heterozygosity was scored. For F-actin staining, embryos were fixed in 8% paraformaldehyde, 0.1 M phosphate buffer (pH 7.4): heptane (1∶1) and hand-peeled for staining with 5 U ml^−1^ Alexa 488-phalloidin (Invitrogen-Molecular Probes). For nuclear staining, Hoescht 33342 was used at either 0.25 µg ml^−1^ for standard immunofluorescence or 1.0 µg ml^−1^ for FISH (Invitrogen-Molecular Probes).

For FISH, 44 oligonucleotide probes, covering the *sry-α* coding sequence were synthesized (Biosearch Technologies), and then labeled with Alexa 488 [43]. Embryos were fixed in 4% paraformaldehyde, 0.1 M phosphate buffer (pH 7.4): heptane (1∶1) for hybridization with 50 µM Alexa 488-*sry-α* probe followed by immunofluorescence staining.

### Image acquisition and analysis

For fixed and living embryos, images were collected on a Zeiss LSM 710 confocal microscope with a 40X/1.2 numerical aperture water-immersion objective (Carl Zeiss, Inc.). Images were collected at a zoom of two, with resolution of 104 nm per pixel.

To segment images, the Image Processing Toolbox in MATLAB (MathWorks) was used. From raw images, the furrow canal network and nuclei masks were generated using a series of morphological operations, as follows. For furrow canal network masks: (1) A Gaussian filter was applied to the raw image, to retain only coarse features of the furrow canals. (2) An intensity threshold was selected manually and applied to generate a preliminary mask. (3) The mask was thinned iteratively until a 1-pixel width network was produced. For nuclei masks: (4) A Gaussian filter was applied to the raw image to smooth out noise, yet retain fine features of the image. A low intensity threshold was selected manually and applied to capture weak furrow canal links present in multinucleated cells. The resulting mask was closed to join disconnected furrow canal links, and thinned iteratively to capture all nuclei separations. Remaining disconnected furrow canal links were removed. (5) The preliminary furrow canal network mask resulting from step 2 above was thinned and added to the mask obtained in step 4. This mask was dilated and inverted to generate a preliminary nuclei mask. Holes inside the mask were filled. (6) Finally, a high threshold was applied to the result of step 1 above; the resulting mask was inverted and then multiplied by the result of step 5 to eliminate boundary artifacts produced by thinning operations.

For quantifications, levels of F-actin in furrow canals and numbers of Amphiphysin tubules were scored as previously reported [29]. The ratio of mononucleate cells to nuclei was generated by manually counting in two quadrants from a raw, single plane, surface view image (quadrant size = 2835 µm^2^) collected at the furrow canals; and the mean was calculated per embryo. The percent of embryos displaying multinucleation was counted manually using raw, single plane, surface view images collected at the furrow canals, where an entire embryo side was visible (≥1500 nuclei assayed per embryo).

For genotyping by FISH, maximum intensity projections were generated from image stacks, comprising ∼4 µm depth and ≥150 nuclei; and active *sry-α* transcription sites were manually counted. Embryos with a maximum of 0, 1, or 2 spots were scored as *sry-α^−/−^*, *sry-α^+/−^*, or *sry-α^+/+^* (wild-type, WT), respectively. For genotyping by immunofluorescence, images were collected at the same microscope settings, and Sry-α signal was scored as either present or absent.

### RT-PCR and western blotting

For RT-PCR, embryos were staged in halocarbon oil 27 under a dissecting microscope. Approximately 100 embryos per stage were homogenized in Trizol (Invitrogen Inc.), and total RNA was extracted in phenol: chloroform (1∶1). Total cDNA was made (SuperScript III First-Strand Synthesis System, Invitrogen), and sequences amplified by PCR. For developmental expression profiles, primers were: *actin42a*-F, *actin42a*-R, *sry-α*-F, *sry-α*-R, *spt*-F, and *spt*-R (for sequences see Table S3). Following RNAi, primers were: *actin42a*-F, *actin42a*-R, *sry-α*-F2, *sry-α*-R2, *spt*-F2, and *spt*-R2 (for sequences see Table S3). Samples were loaded on 1% agarose gels.

For Westerns, 1 hour collections of embryos were incubated at room temperature for 0, 1, 2, 3, 4, or 5 hours respectively. Per stage, 50–100 µl embryos were homogenized in 200 µl 0.05 M Tris pH 8.0, 0.15 M KCl, 0.05M EDTA, 0.5% NP-40, 1X protease inhibitor cocktail (Halt Protease Inhibitor Cocktail, Thermo Scientific). For antibody characterization, 20 adult heads were homogenized in the same buffer. Following a 15 minute spin at 15,000 rpm at 4°C, the cytoplasmic fraction was collected and quantified (Pierce BCA Protein Assay Kit, Thermo Scientific). Equal amounts of total protein were separated on 5–10% SDS-PAGE gels and transferred to nitrocellulose. Sry-α, Spt, HA and α-Tubulin were detected by 1∶5 mouse anti-Sry-α (1G10, Developmental Studies Hybridoma Bank), 1∶500 guinea pig anti-Spt (this paper), 1∶100 rat anti-HA (Roche), and 1∶1000 rat anti-α-Tubulin (T9026, Sigma-Aldrich), respectively. The goat secondary antibodies were HRP conjugates used at 1∶10,000 (Jackson ImmunoResearch).

### RNAi

Approximately 50 pl of *sry-α* and *spt* double-stranded RNA was prepared as previously described [41], with primers: *sry-α*-RNAi-F, *sry-α*-RNAi-R, *spt*-RNAi-F, and *spt*-RNAi-R (for sequences see Table S3), and injected into freshly laid embryos. Following incubation, mitotic cycle 13 embryos were mounted for imaging. RNAi controls were PBS buffer-injected embryos.

### Phylogenetic analysis

Protein sequences were retrieved via UniProt (uniprot.org), and alignments were generated using PROMALS3D (prodata.swmed.edu/promals3d) [37]. Phylogenetic trees were built with PhyML v3.0.1 (pbil.univ-lyon1.fr/software/seaview) [67], and branch supports were tested with the default aLRT SH-like option. In addition, for the small and large trees, bootstrap statistics were determined from 1000 and 500 iterations, respectively. Vinculin from the pre-metazoan *M. brevicollis* served as the outgroup [36]. For both alignments and trees, the same results were generated using either the entire Sry-α and Spt sequences, or the VH2 domain alone. For the roll call analysis, the *D. melanogaster* sequences for *sry-α* and *spt* were used as input for (1) a PSI-BLAST search of the refseq protein database on NCBI (blast.ncbi.nlm.nih.gov); and (2) a BLAST search of the UniProtKB database on UniProt. For those insects where only one paralog was present, we could not assign them as *sry-α* or *spt* based on sequence identity alone because the identity values were the same. Instead, we used the presence or absence of introns as the distinguishing factor: *sry-α* is intronless, whereas *spt* has introns in all Drosophilids. Thus, all insect proteins including introns were assigned as *spt*.

## Supporting Information

Figure S1 Multiple sequence alignment of Vinculin/α-Catenin Superfamily members. Alignment of the VH2 domains (purple) based on secondary structure. Eight α-helices (A–H), corresponding to the repeated four-helix bundles of the α-Catenin M-domain are indicated (gray). Conserved amino acids are in uppercase letters, and symbols are: l, aliphatic (I, V, L); @, aromatic (Y, H, W, F); h, hydrophobic (W, F, Y, M, L, I, V, A, C, T, H); p, polar residues (D, E, H, K, N, Q, R, S, T); t, tiny (A, G, C, S); s, small (A, G, C, S, V, N, D, T, P); b, bulky residues (E, F, I, K, L, M, Q, R, W, Y); c, charged (D, E, K, R, H). Accession numbers are from UniProt or UniParc databases.
(TIF)

Figure S2 Cladogram of Vinculin/α-Catenin Superfamily members. Bootstrap statistics at branch points (500 iterations). Outgroup is *M. brevicollis* Vinculin. Shading highlights division of the clades. This is an expansion of the cladogram shown in Figure 2B. Note that uncharacterized proteins of *D. rerio* and *X. tropicalis* group in the same clade as Sry-α and Spt (F1Q8P1 and F7CCD7, respectively).
(TIF)

Figure S3 RT-PCR of knockdown of mRNAs following RNAi. Transcripts and RNAi treatments listed on the left and top of the gel panels, respectively. *actin42a* is the loading control.
(TIF)

Figure S4 Sry-α and Spt share redundant function during cellularization. (A) Surface views of furrow canals stained for Myosin-2, Sry-α, and Spt-HA (green, red, and blue, respectively), demonstrates how embryos were genotyped for analysis in Figure 4. (B–D) Quantification of multinucleation phenotype following (B) rescue of *sry-α^−/−^* with *sry-α* genomic construct; (C) failure to rescue *nullo^−/−^* with *spt^OE^*; and (D) rescue of *sry-α^−/−^* with *spt^OE^* over a range of temperatures. Each point represents one embryo with ≥150 nuclei analyzed (n = 12 embryos per condition). Note that *spt^OE^* rescue is specific for *sry-α^−/−^* deficiency, and not other actin deficiencies, such as *nullo^−/−^*. Student's t-test was performed to calculate P values as shown in (B, C). Two-way ANOVA analysis was performed to calculate P values as shown in (D).
(TIF)

Figure S5 Characterization of Spt antibody. (A) Spt antibody specifically recognizes endogenous Spt. Spt is not present in adult heads, as reported by publicly available expression data on FlyBase. (B) Spt antibody specifically recognizes endogenous Spt plus Spt-HA in *spt^OE^* embryos. α-Tubulin is the loading control in (A, B).
(TIF)

Figure S6 Embryo genotyping by RNA FISH. (A–C) Images show a representative nucleus (purple) with (A) zero, (B) one, or (C) two *sry-α* transcription sites (green), and the corresponding genotype based on the maximum number of sites seen per nucleus for that embryo. (D) Distribution of embryos assigned to the indicated genotype, based on RNA FISH (n = 148 embryos total; mean ± s.e.m.). The theoretical distribution, according to Mendelian genetics, should be 25% *sry-α^−/−^*; 50% *sry-α^+/−^*; and 25% *sry-α^+/+^* (WT).
(TIF)

Table S1 Roll call analysis of *sry-α* and *spt* in Arthropods and beyond. Accession numbers indicate the presence of *sry-α* and/or *spt* in a given organism. Both *sry-α* and *spt* are present in all Drosophilids, whereas only *spt* is present in the other insects. *D. pulex* and *I. scapularis* are Crustacea and Arachnida, respectively.
(DOC)

Table S2 Antibody concentrations and fixation conditions for immunofluorescence. DSHB, Developmental Studies Hybridoma Bank.
(DOC)

Table S3 Primer sequences. F, forward or sense; R, reverse or antisense.
(DOC)
