# Supplementary figures and images for: The Maternal-to-Zygotic Transition Targets Actin to Promote Robustness during Morphogenesis
Source: PLoS Genet. 2013 Nov 7;9(11):e1003901. doi: 10.1371/journal.pgen.1003901 (PMC3820746; doi:10.1371/journal.pgen.1003901)

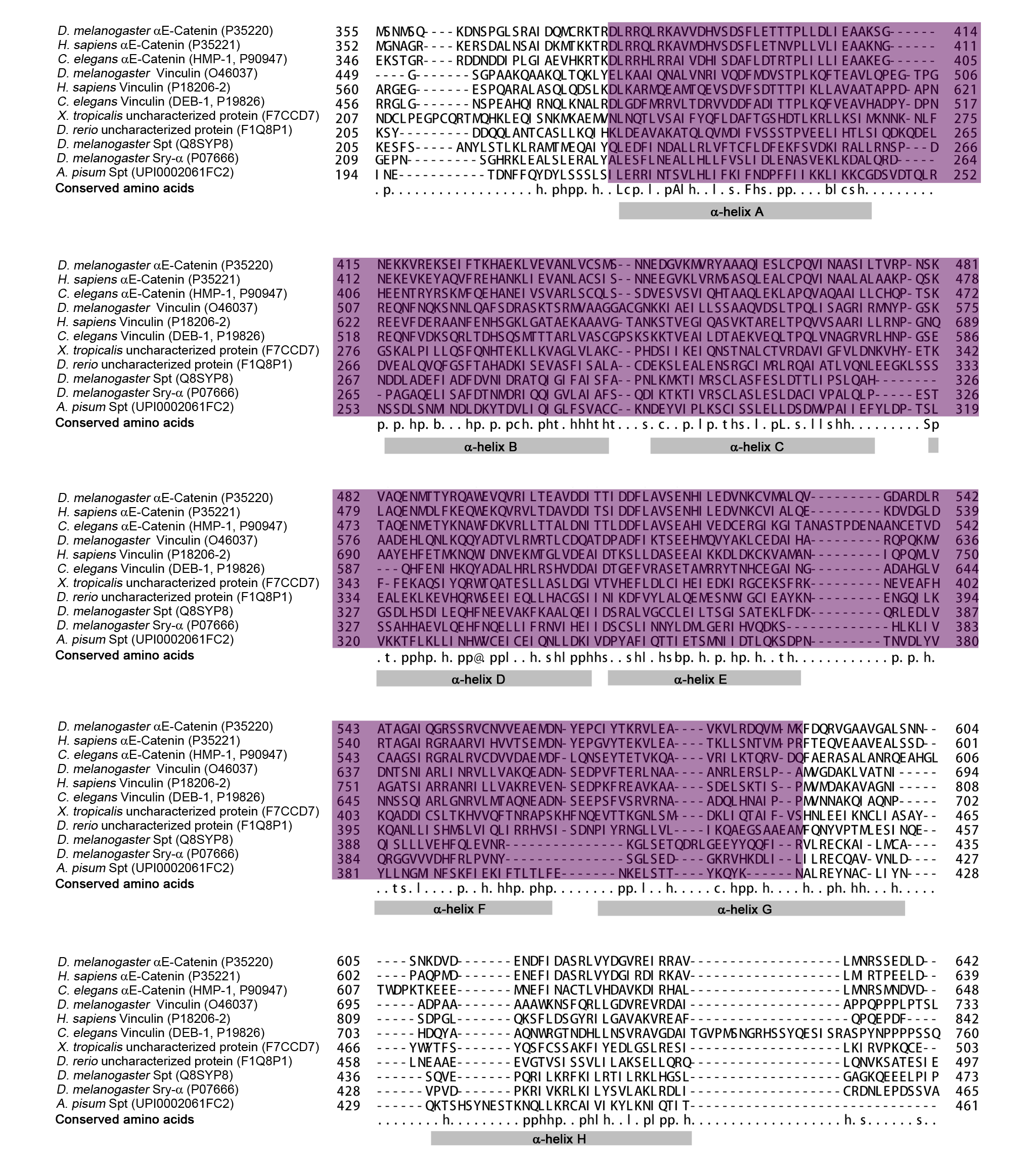

Supplement: Figure S1 — Multiple sequence alignment of Vinculin/α-Catenin Superfamily members. Alignment of the VH2 domains (purple) based on secondary structure. Eight α-helices (A–H), corresponding to the repeated four-helix bundles of the α-Catenin M-domain are indicated (gray). Conserved amino acids are in uppercase letters, and symbols are: l, aliphatic (I, V, L); @, aromatic (Y, H, W, F); h, hydrophobic (W, F, Y, M, L, I, V, A, C, T, H); p, polar residues (D, E, H, K, N, Q, R, S, T); t, tiny (A, G, C, S); s, small (A, G, C, S, V, N, D, T, P); b, bulky residues (E, F, I, K, L, M, Q, R, W, Y); c, charged (D, E, K, R, H). Accession numbers are from UniProt or UniParc databases. (TIF) [file pgen.1003901.s001.tif]

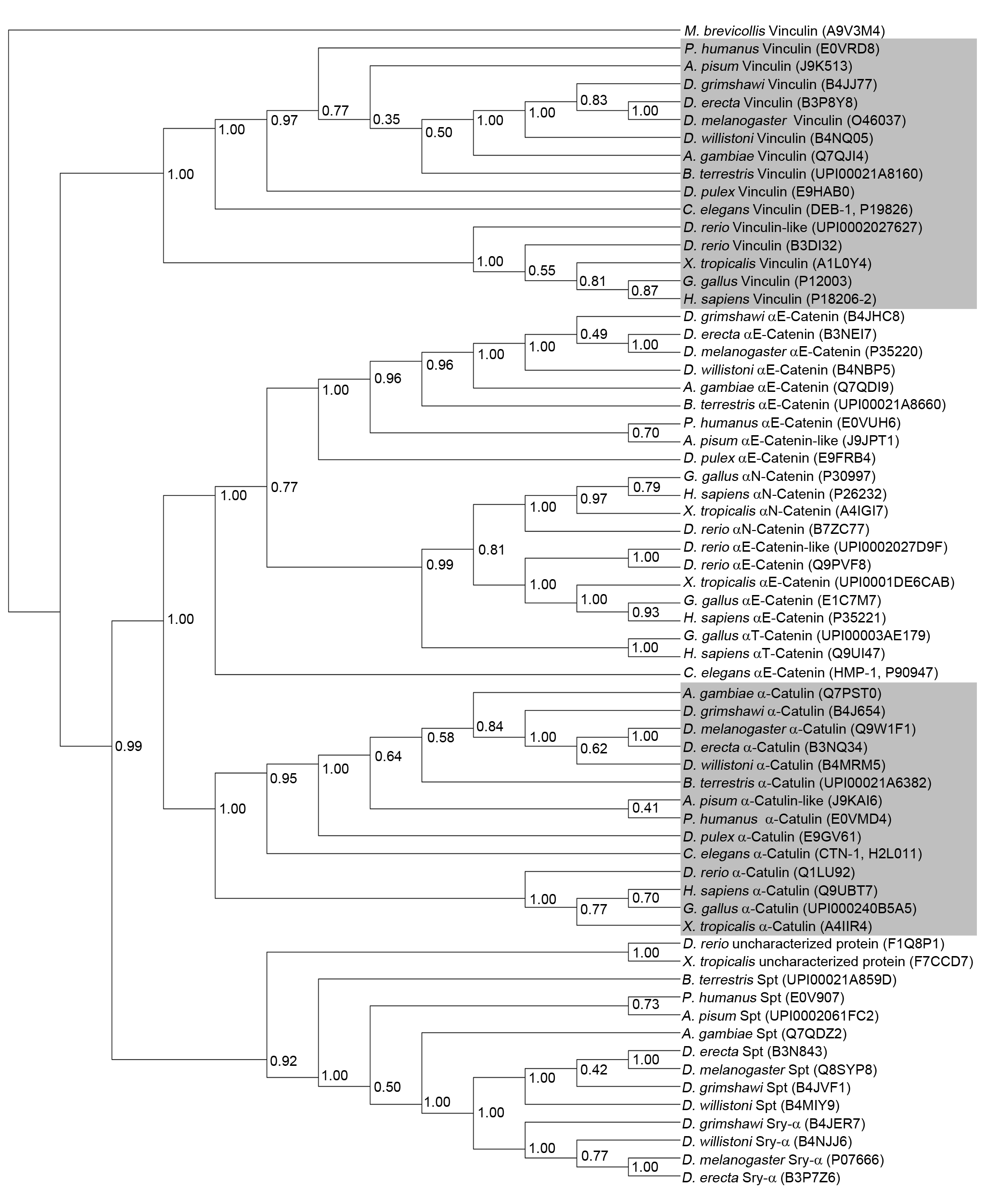

Supplement: Figure S2 — Cladogram of Vinculin/α-Catenin Superfamily members. Bootstrap statistics at branch points (500 iterations). Outgroup is M. brevicollis Vinculin. Shading highlights division of the clades. This is an expansion of the cladogram shown in Figure 2B. Note that uncharacterized proteins of D. rerio and X. tropicalis group in the same clade as Sry-α and Spt (F1Q8P1 and F7CCD7, respectively). (TIF) [file pgen.1003901.s002.tif]

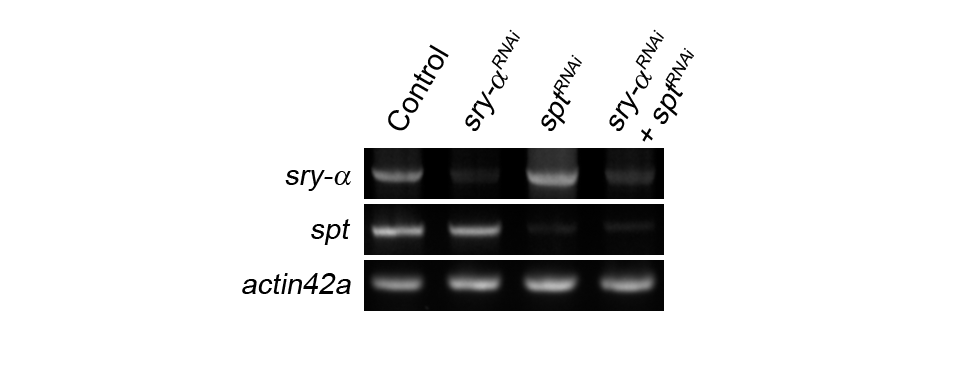

Supplement: Figure S3 — RT-PCR of knockdown of mRNAs following RNAi. Transcripts and RNAi treatments listed on the left and top of the gel panels, respectively. actin42a is the loading control. (TIF) [file pgen.1003901.s003.tif]

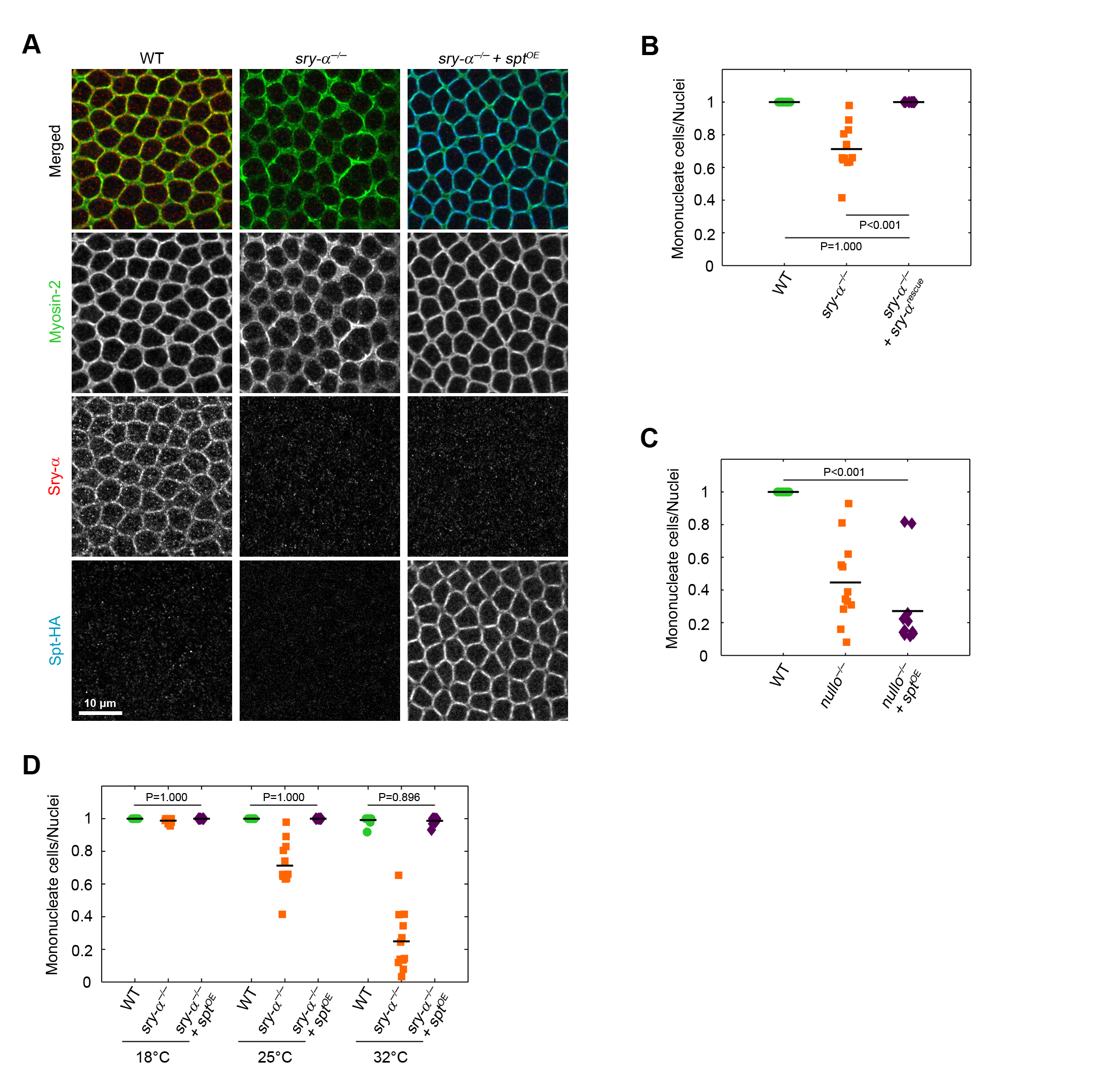

Supplement: Figure S4 — Sry-α and Spt share redundant function during cellularization. (A) Surface views of furrow canals stained for Myosin-2, Sry-α, and Spt-HA (green, red, and blue, respectively), demonstrates how embryos were genotyped for analysis in Figure 4. (B–D) Quantification of multinucleation phenotype following (B) rescue of sry-α−/− with sry-α genomic construct; (C) failure to rescue nullo−/− with sptOE; and (D) rescue of sry-α−/− with sptOE over a range of temperatures. Each point represents one embryo with ≥150 nuclei analyzed (n = 12 embryos per condition). Note that sptOE rescue is specific for sry-α−/− deficiency, and not other actin deficiencies, such as nullo−/−. Student's t-test was performed to calculate P values as shown in (B, C). Two-way ANOVA analysis was performed to calculate P values as shown in (D). (TIF) [file pgen.1003901.s004.tif]

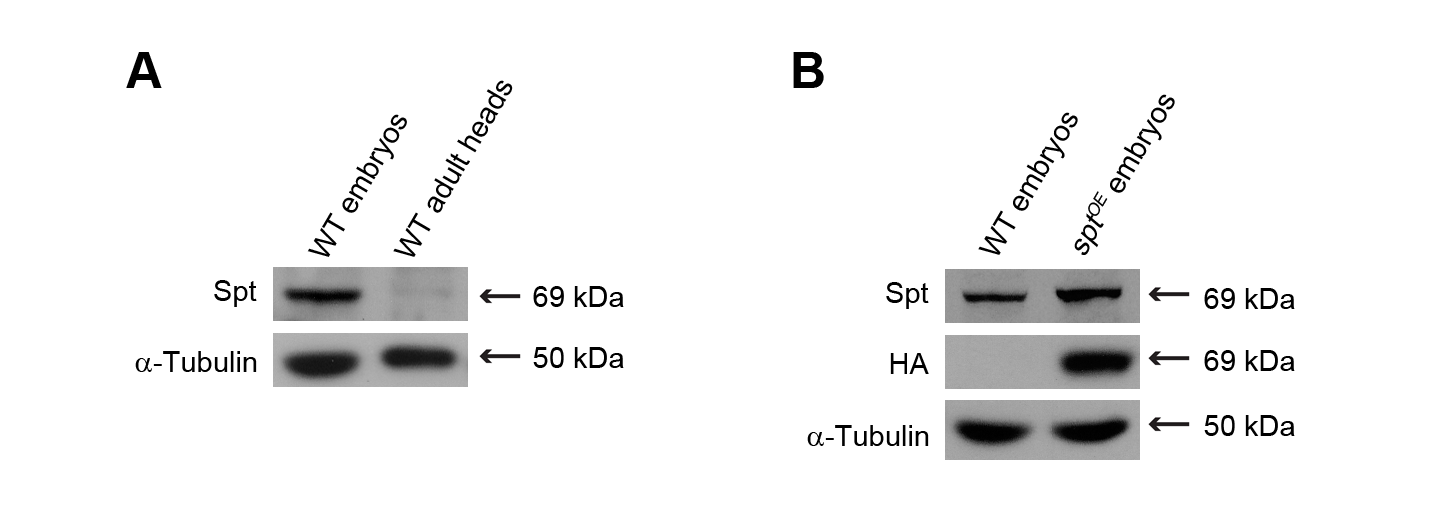

Supplement: Figure S5 — Characterization of Spt antibody. (A) Spt antibody specifically recognizes endogenous Spt. Spt is not present in adult heads, as reported by publicly available expression data on FlyBase. (B) Spt antibody specifically recognizes endogenous Spt plus Spt-HA in sptOE embryos. α-Tubulin is the loading control in (A, B). (TIF) [file pgen.1003901.s005.tif]

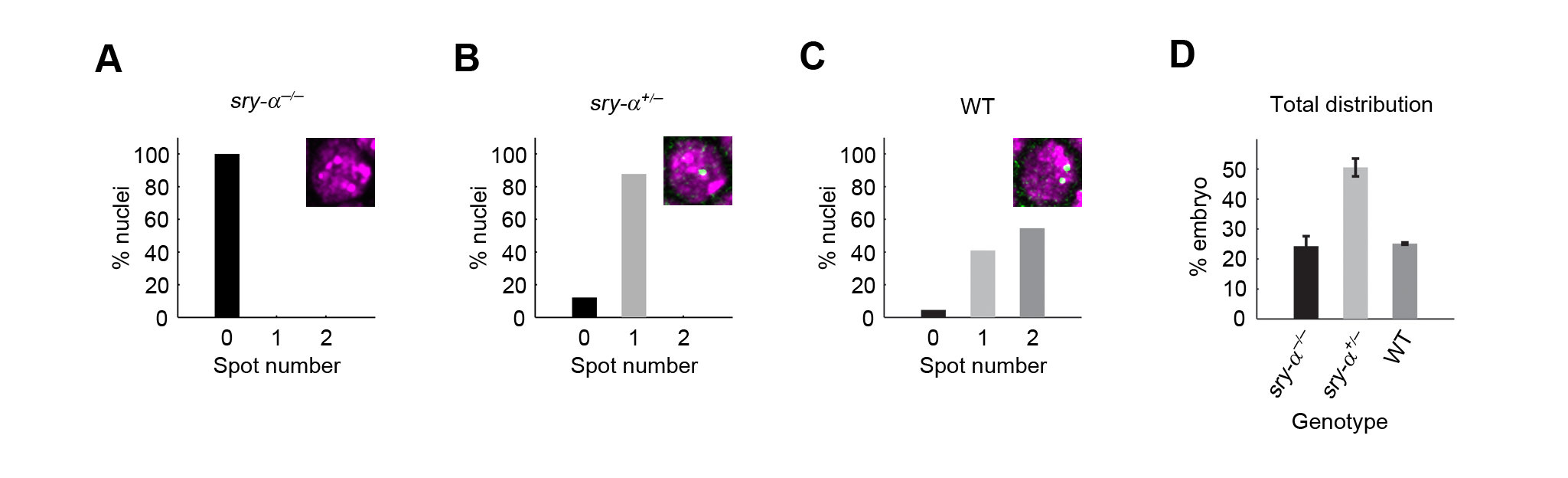

Supplement: Figure S6 — Embryo genotyping by RNA FISH. (A–C) Images show a representative nucleus (purple) with (A) zero, (B) one, or (C) two sry-α transcription sites (green), and the corresponding genotype based on the maximum number of sites seen per nucleus for that embryo. (D) Distribution of embryos assigned to the indicated genotype, based on RNA FISH (n = 148 embryos total; mean ± s.e.m.). The theoretical distribution, according to Mendelian genetics, should be 25% sry-α−/−; 50% sry-α+/−; and 25% sry-α+/+ (WT). (TIF) [file pgen.1003901.s006.tif]
