# Supplementary material for: The Maternal-to-Zygotic Transition Targets Actin to Promote Robustness during Morphogenesis
Source: PLoS Genet. 2013 Nov 7;9(11):e1003901. doi: 10.1371/journal.pgen.1003901 (PMC3820746; doi:10.1371/journal.pgen.1003901)
Supplement: Table S1 — Roll call analysis of sry-α and spt in Arthropods and beyond. Accession numbers indicate the presence of sry-α and/or spt in a given organism. Both sry-α and spt are present in all Drosophilids, whereas only spt is present in the other insects. D. pulex and I. scapularis are Crustacea and Arachnida, respectively. (DOC) [file pgen.1003901.s007.doc]

**Table S1. Presence/absence analysis for *sry-α* and *spt***

| **Organism** | ***sry-α*** | ***spt*** |
| --- | --- | --- |
| ***D. simulans*** | (B4R0Y9) | (B4QGN4) |
| ***D. sechelia*** | (B4HZK9) | (B4HSB5) |
| ***D. melanogaster*** | (P07666) | (Q8SYP8) |
| ***D. yakuba*** | (B4PLR5) | (B4P3E4) |
| ***D. erecta*** | (B3P7Z6) | (B3N843) |
| ***D. ananassae*** | (B3MSV2) | (B3ME95) |
| ***D. pseudoobscura*** | (Q07964) | (Q28Y82) |
| ***D. persimilis*** | (B4GNH9) | (B4GCU5) |
| ***D. willistoni*** | (B4NJJ6) | (B4MIY9) |
| ***D. mojavensis*** | (B4K800) | (B4KTQ2) |
| ***D. virilis*** | (O77201) | (B4LNP0) |
| ***D. grimshawi*** | (B4JER7) | (B4JVF1) |
| ***A. aegypti*** | - | (Q17MT4) |
| ***C. quinquefasciatus*** | - | (B0W8C8) |
| ***A. gambia*** | - | (Q7QDZ2) |
| ***A. mellifera*** | - | (H9KK85) |
| ***B. terrestris*** | - | (UPI00021A859D) |
| ***N. vitripennis*** | - | (K7JEY5) |
| ***H. saltator*** | - | (E2BCZ7) |
| ***S. invicta*** | - | (E9IZK9) |
| ***A. cephalotes*** | - | (H9I229) |
| ***C. floridanus*** | - | (E2AWT2) |
| ***T. castaneum*** | - | (D2A524) |
| ***P. humanus*** | - | (E0V907) |
| ***A. pisum*** | - | (UPI0002061FC2) |
| ***D. plexippus*** | - | (G6DSZ2) |
| ***D. pulex*** | - | - |
| ***I. scapularis*** | - | - |
| ***C. elegans*** | - | - |
| ***M. brevicollis*** | - | - |
