# Supplementary material for: The Maternal-to-Zygotic Transition Targets Actin to Promote Robustness during Morphogenesis
Source: PLoS Genet. 2013 Nov 7;9(11):e1003901. doi: 10.1371/journal.pgen.1003901 (PMC3820746; doi:10.1371/journal.pgen.1003901)
Supplement: Table S2 — Antibody concentrations and fixation conditions for immunofluorescence. DSHB, Developmental Studies Hybridoma Bank. (DOC) [file pgen.1003901.s008.doc]

**Table S2. Antibody concentrations for immunofluorescence**

| **Antibody** | **Fixation** | **Concentration** | **Source** |
| --- | --- | --- | --- |
| **Rabbit anti-Myosin-2 (Zipper)** | Heat or Formaldehyde | 1:1000 | Sokac Lab |
| **Rat anti-HA** | Heat or Formaldehyde | 1:50 | 11867423001-Roche |
| **Mouse anti-Sry-α** | Formaldehyde | 1:5 | 1G10-DSHB |
| **Mouse anti-Peanut** | Formaldehyde | 1:200 | 4C9H4-DSHB |
| **Rabbit anti-Amphiphysin** | Formaldehyde | 1:1000 | Gift of G. Boulianne |
| **Goat anti-Mouse, Rabbit, or Rat**  **Alexa-488** | Heat or Formaldehyde | 1:500 | Invitrogen-Molecular Probes |
| **Goat anti-Mouse, Rabbit, or Rat Alexa-568** | Heat or Formaldehyde | 1:500 | Invitrogen-Molecular Probes |
