# Supplementary material for: The Maternal-to-Zygotic Transition Targets Actin to Promote Robustness during Morphogenesis
Source: PLoS Genet. 2013 Nov 7;9(11):e1003901. doi: 10.1371/journal.pgen.1003901 (PMC3820746; doi:10.1371/journal.pgen.1003901)
Supplement: Table S3 — Primer sequences. F, forward or sense; R, reverse or antisense. (DOC) [file pgen.1003901.s009.doc]

**Table S3. Primer sequences for RT-PCR and RNAi**

| **Primer** | **Sequence** |
| --- | --- |
| ***actin42a*-F** | 5'-TTGGACTTCGAGCAGGAGAT-3' |
| ***actin42a*-R** | 5'-AATCTTCATGGTGGACGGAG-3' |
| ***sry-α-*F** | 5'-AACATTTCTGTTTCCCGGAG-3' |
| ***sry-α-*R** | 5'-GCCAGCTGGTAGGTGTCTTC-3’ |
| ***sry-α*-F2** | 5'-CCGCTCGAGCCATGGAACAGCTATTGGC-3' |
| ***sry-α*-R2** | 5'-CCGGAATTCTCAATCTAACCTAAGAATCTCA-3’ |
| ***sry-α*-RNAi-F** | 5'- TAATACGACTCACTATAGGGTCAGGAGCTAATC-3' |
| ***sry-α*-RNAi-R** | 5'- TAATCAGACTCACTATAGGGCCCAGCATGTCCA-3’ |
| ***spt*-F** | 5’-AAATGGAAATTTTGCGAACG-3’ |
| ***spt*-R** | 5’-TCTGGCTGAGTGGAAGGACT-3’ |
| ***spt*-F2** | 5’-CCGCTCGAGACCATGGAAATTTTGCGAACG-3’ |
| ***spt*-R2** | 5’-CGCGGATCCTCAGCATTCAAAGTATCC-3’ |
| ***spt*-RNAi-F** | 5’-TAATACGACTCACTATAGGGGTTGGTTGTTGCC-3’ |
| ***spt*-RNAi-R** | 5’-TAATACGACTCACTATAGGGTCGTAGAGGATAC-3’ |
